# Prognostic significance of the systemic immune-inflammation index in patients with extranodal natural killer/T-cell lymphoma

**DOI:** 10.3389/fonc.2023.1273504

**Published:** 2023-10-16

**Authors:** Tao Hai, Wanchun Wu, Kexin Ren, Na Li, Liqun Zou

**Affiliations:** ^1^ Division of Medical Oncology, Cancer Center and State Key Laboratory of Biotherapy, Sichuan University West China Hospital, Chengdu, China; ^2^ Department of Hematology-Oncology, Chongqing Key Laboratory of Translational Research for Cancer Metastasis and Individualized Treatment, Chongqing University Cancer Hospital, Chongqing, China

**Keywords:** systemic immune-inflammation index, extranodal natural killer/T-cell lymphoma, nomogram, prognosis, risk group

## Abstract

**Background:**

The systemic immune-inflammation index (SII) is based on the neutrophil, platelet, and lymphocyte counts, and has been identified as a prognostic marker in multiple types of cancer. However, the potential value of the SII for predicting survival outcomes in patients with extranodal natural killer/T-cell lymphoma (ENKTCL) has not been investigated thus far.

**Method:**

This study included 382 patients with ENKTCL treated with asparaginase-base regimens from 2021 to 2017 in West China Hospital (Chengdu, China). Clinical and demographic variables, as well as the prognostic value of the SII, were analyzed using Cox proportional hazards regression analysis.

**Results:**

The complete and objective response rates were 55.8% and 74.9%, respectively. Patients with high SII were associated with a lower rate of complete response, higher rate of B symptoms, and serum lactate dehydrogenase levels above or equal to the upper limits of normal (*p* < 0.01). Patients with low SII were linked to better overall survival and progression-free survival than those with high SII (*p* < 0.01). Patients with early-stage disease or prognostic model for natural killer lymphoma with Epstein–Barr virus, defined as the low-risk group, could be further stratified according to the SII (*p* < 0.01). Negative prognostic factors were determined using the Cox proportional hazards regression analysis, which identified four variables: Eastern Cooperative Oncology Group performance status score ≥2, Stage III/IV disease, positivity for Epstein–Barr virus DNA in plasma, and high SII. Predictive nomograms for the prediction of 3- and 5-year overall survival, as well as progression-free survival, were constructed based on those four variables. The nomograms demonstrated favorable discriminating power.

**Conclusion:**

The SII is a novel prognostic marker for ENKTCL, which may be used for the prediction of poorer survival in low-risk patients.

## Introduction

Extranodal natural killer/T-cell lymphoma (ENKTCL) is a unique hematological malignancy universally characterized by invasion of the nasal cavity and Epstein–Barr virus (EBV) infection among a variety of lymphoma subtypes (1). The disease is highly prevalent in the Asia-Pacific region and Latin America, accounting for nearly 10% of newly diagnosed cases of non-Hodgkin lymphoma in those regions. However, this malignancy is remarkably rare in Europe and North America (2, 3). The typical pathological features of ENKTCL include negativity for surface CD3 and positivity for biomarkers such as CD3 epsilon (CD3ϵ), CD56, cytotoxic molecules (perforin, granzyme B, and T-cell-restricted intracellular antigen-1 [TIA-1]), and EBV-encoded small RNA (EBER) detectable by *in situ* hybridization (ISH) (4).

In the past two decades, advances in treatment have drastically improved the overall survival (OS) of patients with ENKTCL. Concurrent chemoradiotherapy (CCRT) or sequential chemoradiotherapy (SCRT) with asparaginase-base regimens that include L-asparaginase is the optimal strategy for the treatment of early-stage localized disease (5). For advanced-stage or relapsed/refractory ENKTCL, the use of intensive chemotherapeutic regimens, such as SMILE (i.e., dexamethasone, methotrexate, ifosfamide, L-asparaginase, and etoposide) or DDGP (i.e., dexamethasone, cisplatin, gemcitabine, and pegaspargase), is recommended; nevertheless, severe bone marrow suppression and infection may occasionally lead to considerable treatment-related mortality (6–8).

Although early-stage ENKTCL is associated with relatively good prognosis, the 5-year OS rate among patients with advanced disease was 50% (7). Predictive models focusing on the prognostic factors associated with survival in ENKTCL were developed to stratify patients into different groups based on their risk. These models include the International Prognostic Index (IPI) and the Korean Prognostic Index during the anthracycline era (9, 10), and the prognostic model for natural killer lymphoma with Epstein–Barr virus (PINK-E) in the non-anthracycline era (11). The nomogram-revised risk index developed by a Chinese oncologist demonstrated a better performance for the stratification of patients with early-stage disease than other models (IPI, Korean Prognostic Index, and PINK-E) (12). Evidence has revealed that the inflammatory microenvironment plays a major role in the development of cancer (13, 14). A novel predictive tool, termed the systemic immune-inflammation index (SII), is calculated based on the counts of inflammatory cells, namely, neutrophils, platelets, and lymphocytes. This tool was identified as a prognostic factor in a variety of malignancies (15). Studies showed that gastric cancer and metastatic renal cell cancer patients with low SII were linked to longer disease-free survival and OS (15, 16).

In this study, we initially evaluated the prognostic value of the SII in patients with ENKTCL receiving treatment with asparaginase-containing regimens. A predictive nomogram based on the SII and other significantly valuable variables was constructed to assist clinical physicians in the stratification of patients with ENKTCL.

## Materials and methods

### Patient collection

In this retrospective study, we reviewed patients diagnosed with ENKTCL in the West China Hospital of Sichuan University (Chengdu, China) from 2017 to 2021. For all patients, the diagnosis was strictly confirmed by a senior pathologist, and the disease was staged according to the Ann Arbor staging system. The inclusion criteria were as follows: (1) diagnosis of ENKTCL according to the World Health Organization classification of lymphoid neoplasms (17); (2) age ≥18 years; (3) no apparent evidence of other malignancies; (4) availability of complete clinical data; and (5) use of L-asparaginase- or pegaspargase-containing regimens as first-line treatment. This study was approved by the research ethics committee of the West China Hospital (approval number: SCHX-2022-64).

Clinicopathological characteristics included age, sex, Eastern Cooperative Oncology Group performance status (ECOG-PS) score, B symptoms, Ann Arbor stage, nasal type, plasma EBV-DNA, lactate dehydrogenase (LDH) levels, and PINK-E score. Pretreatment peripheral venous blood parameters (collected within 1 week before the initiation of first-line treatment) included the counts of neutrophils, lymphocytes, and platelets. The data of all variables mentioned above were utilized in the analysis.

### SII score

Peripheral venous blood parameters were used to calculate the SII based on the following formula: SII = platelets (P) × neutrophils (N)/lymphocytes (L). Subsequently, the receiver operating characteristic (ROC) curve was plotted to determine the optimal cutoff value for the SII. Next, the patients were divided into two groups according to their SII: low SII score (SII-L) and high SII score (SII-H). Differences in the clinical features of these two groups were assessed.

### Treatment

All patients received at least one cycle of chemotherapy combined with CCRT or SCRT. All patients received asparaginase-based chemotherapy as initial treatment; the regimens included the following: VDLP (L-asparaginase, cisplatin, etoposide, and dexamethasone); VLP (L-asparaginase, vincristine, and prednisone); GLIDE (gemcitabine, L-asparaginase, ifosfamide, dexamethasone, and etoposide); or GEMOX (pegaspargase, gemcitabine, and oxaliplatin). The radiotherapy dose was 50 Gy at the involved field, with 1.8–2.0 Gy per fraction for a total of five fractions per week.

Patients with early-stage ENKTCL received chemotherapy combined with involved-field radiation therapy (CCRT or SCRT) after initial diagnosis. Patients with advanced-stage ENKTCL primarily received L-asparaginase-containing chemotherapy. Moreover, residual lesions detected by positron emission tomography/computed tomography could be managed with consolidative radiotherapy depending on the preference of the treating physician. Hematopoietic stem cell transplantation (HSCT) was performed for patients who achieved complete response (CR) or partial response (PR); however, its clinical significance remains controversial.

### Efficacy evaluation

Treatment responses were classified based on recommendations for the initial evaluation, staging, and response assessment of Hodgkin and non-Hodgkin lymphoma, namely, the Lugano classification (18); this classification includes CR, PR, stable disease, and progressive disease (PD). The overall response rate was calculated as the percentage of patients achieving either CR or PR. OS was determined as the time period from initial diagnosis to the last follow-up or death by any cause. Progression-free survival (PFS) was determined as the time period between initial diagnosis and disease recurrence, disease progression, last follow-up, or death.

### Statistical analysis

The ratio closest to the sum value of maximum sensitivity and specificity was regarded as the optimal cutoff value for SII, as determined using the ROC curve. The optimal cutoff value of SII was 601×10^9^ cells/L (specificity: 0.713; sensitivity: 0.474; ROC: 0.578; 95% confidence interval: 0.520–0.635) (**Supplementary Figure 1**). Continuous variables are presented as median number and range, while categorical variables are presented as frequency with percentage. Differences in variables between the SII-L and SII-H groups were analyzed through the chi-squared test. The OS and PFS were described using Kaplan–Meier survival curves, and the log-rank test was performed to compare discrepancies in survival. All statistical analyses and curve plotting were performed using R software (version 4.2.2). *p*-values < 0.01 indicate statistically significant differences.

## Results

### Patient characteristics

Following exclusion, a total of 382 patients were assigned to the SII-L and SII-H groups. The characteristics of all patients are presented in **Table 1**. Most patients were men (65.4%), and more than half (51.6%) experienced B symptoms. Among the patients, 110 (28.8%) and 330 (86.4%) had stage III/IV disease and nasal type lymphoma, respectively. Laboratory analyses revealed that the mean counts of neutrophils, platelets, and lymphocytes were 13.28, 201.32, and 1.30 ×10^9^/L, respectively. The majority of patients (72.5%) exhibited positivity for EBV-DNA in the plasma, while 59.7% had LDH levels above or equal to the upper limits of normal (ULN). According to the PINK-E model, 262 patients (68.6%) were assigned to the lower-risk group (0–1). In terms of differences between groups, the SII-L group included fewer patients than the SII-H group (148 [38.7%] vs. 234 [61.3%], respectively). The median age in both groups was 44 years (range: 18–77 years and 12–79 years, respectively).

**Table 1:** Clinical characteristics of enrolled ENKTL patients divided by SII.

Variables	All patients (*N* = 382) (%)	SII-L (*N* = 148) (%)	SII-H (*N* = 234) (%)	*p*-value
Age (years)				
Median(range)	44 (12–79)	44 (18–77)	44 (12–79)	0.051
≤60	320 (83.8)	116 (78.4)	204 (87.2)	0.094
>60	62 (16.2)	32 (21.6)	30 (12.8)	
Sex				
Male	250 (65.4)	92 (62.2)	158 (67.5)	0.430
Female	132 (34.6)	56 (37.8)	76 (32.5)	
ECOG score				
0 or 1	351 (91.9)	138 (93.2)	213 (91.0)	0.602
≥2	31 (8.1)	10 (6.8)	21 (9.0)	
B symptom				
Presence	197 (51.6)	58 (39.2)	139 (59.4)	0.005
Absence	185 (48.4)	90 (60.8)	95 (40.6)	
Stage				
I or II	272 (71.2)	114 (77.0)	158 (67.5)	0.126
III or IV	110 (28.8)	34 (23.0)	76 (32.5)	
Nasal				
Yes	330 (86.4)	132 (89.2)	198 (84.6)	0.400
No	52 (13.6)	16 (10.8)	36 (15.4)	
Plasma EBV-DNA				
Positive	277 (72.5)	97 (65.5)	180 (76.9)	0.068
Negative	105 (27.5)	51 (34.5)	54 (23.1)	
PINK-E				
0 or 1	262 (68.6)	109 (73.6)	153 (65.4)	0.167
≥2	120 (31.4)	39 (26.4)	81 (34.6)	
Serum LDH				
<ULN (250U/L)	228 (59.7)	113 (76.4)	115 (49.1)	<0.001
≥ULN (250U/L)	154 (40.3)	35 (23.6)	119 (50.9)	

SII-L, systemic immune-inflammation index-low; SII-H, systemic immune-inflammation index-high; ECOG, Eastern Cooperative Oncology Group; EBV, Epstein–Barr virus; PINK-E, prognostic index of natural killer lymphoma with Epstein–Barr virus; LDH, lactate dehydrogenase.

### Differences between the SII-L and SII-H groups

In this study, the CR and overall response rates of all patients were 55.8% and 74.9%, respectively. The rate of CR was significantly higher among patients in the SII-L group versus the SII-H group (67.6% vs. 48.3, respectively; *p* = 0.004); however, there were no differences observed in PR, stable disease, and PD between the two groups (*p* = 0.246, *p* = 0.054, and *p* = 0.172, respectively) (**Supplementary Table 1**). Furthermore, we observed significant differences between the SII-L and SII-H groups in B symptoms and serum LDH levels (*p* < 0.001). There was no correlation found in any of the other variables between the SII-L and SII-H groups (*p* > 0.01) (**Table 1**).

### Long-term follow-up of patients with ENKTCL

**Figure 1:**
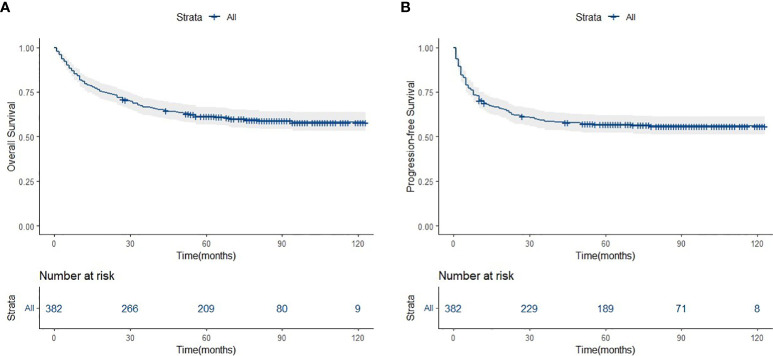
Overall survival **(A)** and progression-free survivsl **(B)** of asparaginase-based chemotheraphy for ENKTCL patients.

**Figure 2:**
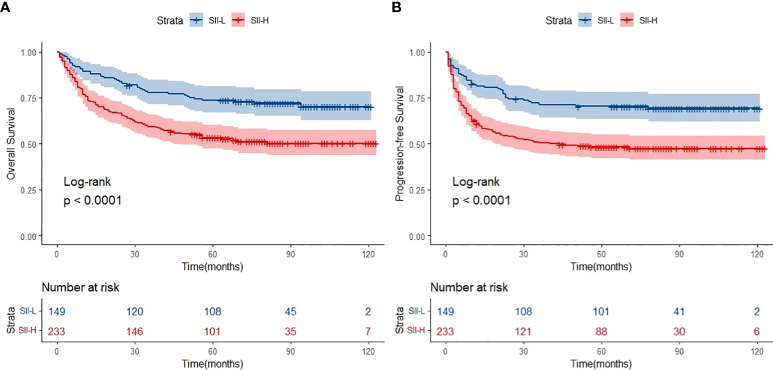
Overall survival (OS) **(A)** and progression-free survival (PFS) **(B)** of SII-L and SII-H groups in ENKTL patients.

**Figure 3:**
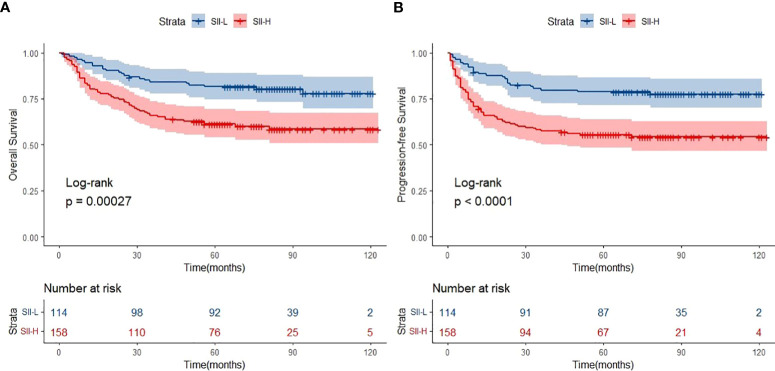
Overall survival (OS) **(A)** and progression-free survival (PFS) **(B)** of SII-L and SII-H groups in early stage KTCL patients.

The median follow-up period was 62.0 months (range: 1.0–123.0 months). A total of 154 deaths and 166 cases of disease progression were recorded. In addition, 80 and 69 patients experienced PD and disease recurrence, respectively, after initial treatment. Seven of them underwent HSCT (auto-HSCT, *n* = 5; allogenic-HSCT, *n* = 2) after receiving treatment with the SMILE regimen. Regarding survival, the 5-year OS and PFS rates were 61.4% and 56.9%, respectively (**Figure 1**). Patients in the SII-L group had better OS and PFS than those in the SII-H group (5-year OS: 73.6% vs. 53.6%, *p* < 0.0001; 5-year PFS: 70.8% vs. 48.0%, *p* < 0.0001) (**Figure 2**). Among patients with early-stage (I/II) disease, better OS and PFS were observed in those with SII-L versus SII-H (5-year 81.5% vs. 61.2%, *p* = 0.00027; 5-year PFS: 78.8% vs. 55.5%, *p* < 0.0001) (**Figure 3**). However, these differences were not confirmed in patients with advanced-stage (III/IV) disease (OS, *p* = 0.2; PFS, *p* = 0.29) (**Supplementary Figure 2**).

### Prognostic factors and nomogram construction

The univariate Cox proportional hazards regression analysis identified eight variables that were related to OS (*p* < 0.01): ECOG-PS, B symptoms, disease stage, non-nasal disease, positivity for EBV-DNA in plasma, LDH levels, PINK-E score, and SII. The univariate analysis for PFS yielded similar results, with the exception of B symptoms (*p* = 0.018) (**Table 2**). The multivariate Cox proportional hazards regression analysis identified only four variables significantly associated with OS and PFS (*p* < 0.01): ECOG-PS; stage; positivity for EBV-DNA in plasma; and SII (**Table 2**).

**Table 2:** Univariate (A) and multivariate (B) Cox regression analysis of progression-free survival and overall survival in ENKTL patients.

Variables	Progression-free survival		Overall survival	
	OR (95% CI)	*p-*value	OR (95% CI)	*p-*value
A				
**Age >60**	0.924 (1.083–1.404)	0.720	1.238 (0.859–1.783)	0.252
**Sex male**	1.221 (0.819–1.693)	0.241	1.076 (0.821–1.412)	0.596
**ECOG score ≥2**	2.582 (1.646–4.051)	<0.001	3.684 (2.411–5.630)	<0.001
**B symptoms**	1.445 (1.062–1.966)	0.018	1.805 (1.303–2.502)	<0.001
**Stage III/IV**	2.496 (1.831–3.401)	<0.001	2.623 (1.907–3.609)	<0.001
**Non-nasal**	2.332 (1.604–3.391)	<0.001	2.541 (1.734–3.724)	<0.001
**Plasma EBV-DNA**	2.206 (1.477–3.295)	<0.001	3.697 (2.258–6.051)	<0.001
**LDH≥ULN (250 U/L)**	1.632 (1.203–2.215)	0.002	1.855 (1.351–2.548)	<0.001
**PINK-E score ≥2**	2.286 (1.680–3.109)	<0.001	2.582 (1.880–3.547)	<0.001
**SII-H**	2.082 (1.477–2.933)	<0.001	2.067 (1.448–2.950)	<0.001
B				
**ECOG score ≥2**	1.770 (1.113–2.814)	0.016	2.530 (1.630–3.928)	<0.001
**B symptoms**	1.268 (0.923–1.743)	0.143	1.228 (0.873–1.728)	0.239
**Stage III/IV**	2.096 (1.523–2.886)	<0.001	2.043 (1.467–2.845)	<0.001
**Non-Nasal**	1.566 (1.035–2.370)	0.033	1.277 (0.811–2.010)	0.290
**Plasma EBV-DNA**	1.880 (1.254–2.818)	0.002	3.178 (1.937–5.214)	<0.001
**LDH≥ULN (250 U/L)**	1.262 (0.910–1.750)	0.163	0.973 (1.028–1.385)	0.878
**PINK-E score ≥2**	1.048 (0.637–1.723)	0.854	1.067 (0.645–1.765)	0.801
**SII-H**	1.827 (1.293–2.580)	<0.001	1.787 (1.250–2.553)	0.001

CI, confidence interval; SII-L, systemic immune-inflammation index-low; SII-H, systemic immune-inflammation index-high; EBV, Epstein–Barr virus; ECOG, Eastern Cooperative Oncology Group performance status; OR, odds ratio; LDH, lactate dehydrogenase; PINK-E, prognostic index of natural killer lymphoma with Epstein–Barr virus.

**Figure 4:**
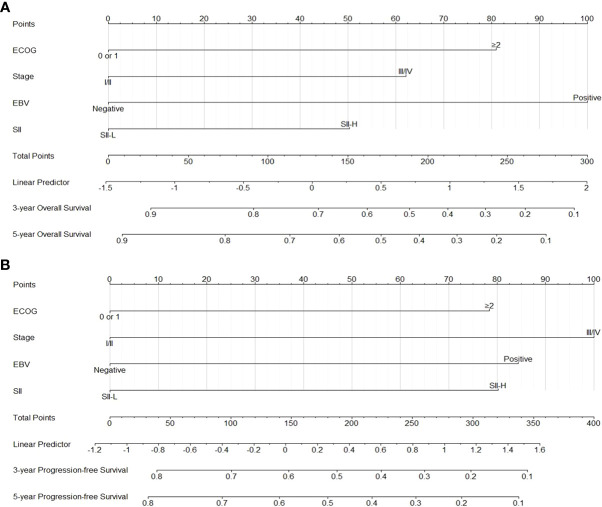
Nomograms for predicting 3-5 year overall survival **(A)** and 3-5 yearprogression-free survival **(B)**.

Following Cox proportional hazards regression analysis, it was concluded that ECOG-PS score ≥2, stage III/IV disease, positivity for EBV-DNA in plasma, and SII-H were negatively associated with the OS and PFS of patients with ENKTCL. Based on these four prognostic factors, two nomograms were constructed for the prediction of OS and PFS in patients with ENKTCL (**Figure 4**). The nomograms were validated and calibrated using the bootstrap method and calibration curves (**Supplementary Figure 3**). The concordance index of the nomograms for predicting the probabilities of 3- and 5-year OS and PFS was 0.71 and 0.68, respectively, demonstrating a favorable predictive power.

### Further risk stratification based on the SII in addition to PINK-E

**Figure 5:**
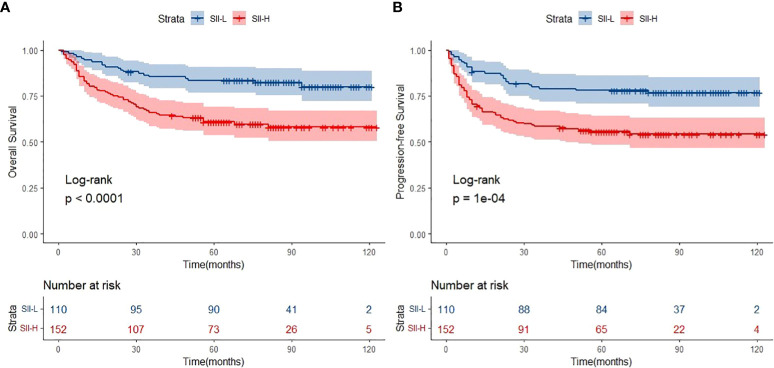
Overall survival (OS) **(A)** and progression-free survival (PFS) **(B)** of SII-L and SII-H groups in low-risk patients defined by prognostic index of natural killer lymphoma with Epstein-Barr virus (PINK-E).

We found that SII-H was a negative prognostic factor for OS and PFS. Thus, we sought to further stratify the patients by adding the SII into the PINK-E model. Based on this approach, 262, 83, and 37 patients were classified into the low-, intermediate-, and high-risk groups, respectively. In the low-risk group, OS and PFS were significantly lower in patients with SII-L than in those with SII-H (**Figure 5**). In contrast, in the intermediate- and high-risk groups, we did not observe any significant difference in OS and PFS between patients with SII-L and those with SII-H (OS, *p* = 0.16 vs. 0.51, respectively; PFS, *p* = 0.087 vs. 0.16, respectively) (**Supplementary Figures S4, S5**).

## Discussion

In lymphoma, pyrexia is a paraneoplastic effect thought to be associated with the release of cytokines such as IL-6 and TNF-α, and those cytokines were manifestations of tumor-related inflammation. Hence, we decided to explore the potential prognostic value of SII among ENKTCL as it has not been studied before. In this large, real-world, retrospective study, treatment of patients with ENKTCL using L-asparaginase-based regimens resulted in satisfactory short- and long-term efficacy. In addition, this was the first investigation evaluating the prognostic value of the SII in ENKTCL. Based on the results obtained from the Cox proportional hazards regression analysis, ECOG-PS score ≥2, stage III/IV disease, positivity for EBV-DNA in plasma, and SII-H were associated with worse OS and PFS. Furthermore, the SII was valuable in predicting the survival of patients with early-stage ENKTCL, and it could be used to further identify ENKTCL patients with better OS and PFS in the low-risk group based on the PINK-E model. Finally, we developed prognostic nomograms to predict 3- and 5-year OS and PFS with favorable precision based on the factors mentioned above.

Large-scale, retrospective, long-term follow-up studies of ENKTCL patients treated with asparaginase-base regimens have revealed significant improvements in survival. Kim et al. conducted a large multicenter study, which involved 527 patients with ENKTCL. The rates of 3-year OS and PFS were 59% and 48%, regardless of the risk group (11). Similarly, another multicenter study conducted by Yamaguchi et al. reported the survival outcomes for 358 ENKTCL patients treated with asparaginase-base regimens. The 5-year OS and PFS rates in that study were 56% and 45%, respectively (18). In a multicenter study including 166 patients with ENKTCL, Fox et al. reported 5-year OS and PFS of 50% and 42%, respectively (19).

Systemic inflammation occurring during the development and progression of cancer has been recognized as a result of local immune response at the tumor site (13, 14, 20). Basic research revealed the potential role of hemocytes during systemic inflammation. Labelle et al. reported that C-X-C motif chemokine ligand 5 (CXCL5) and CXCL7 chemokines secreted by platelets were essential elements for the rapid recruitment of granulocytes to tumor cells to form “early metastatic niches” (21). Lymphocytes are the key cells in the immune surveillance of tumors. Tumor-infiltrating lymphocytes migrate from peripheral blood to the tumor microenvironment, which has been associated with increased programmed cell death-ligand 1 (PD-L1) infiltration. Theoretically, this process increases the efficacy of immune checkpoint inhibitors (22, 23). In addition, Xian et al. found that the neutrophil-associated inflammatory microenvironment aids tumor cell mobility and promotes breast-to-lung metastasis by the recruitment of neutrophils (24). On a large scale, the alterations in blood cell parameters unveiled numerous interactions between tumor cells and inflammatory or immune cells in tumor microenvironments. These findings indicate that the combination of platelets, neutrophils, and lymphocytes may be a prognostic factor for patients with tumors. The SII has been associated with survival in multiple malignancies. He et al. reported that SII-L in elderly patients with stage-II gastric cancer was linked to significantly better 5-year OS versus SII-H (92% vs. 80%, respectively) (25). Huang et al. showed that SII-H was associated with poor prognosis in patients with cervical cancer, and area under the curve for multiple prognostic parameters demonstrated that the SII was effective in predicting 3- and 5-year survival (26). Studies also evaluated the prognostic value of the SII in hematolymphoid tumors. In patients with classical Hodgkin lymphoma, the SII was a better prognostic factor compared with the neutrophil-to-lymphocyte ratio, prognostic nutritional index, and beta-2-microglobulin (B2M) (27). Wang et al. discovered that diffuse large B-cell lymphoma patients with SII-H tended to have higher levels of LDH, more advanced-stage disease, poor PS, and high IPI score versus those with SII-L (28). To the best of our knowledge, this is the first large-scale retrospective study focusing on the SII in patients with ENKTCL.

Firstly, the primary results of the present study were compatible with those of previous studies. The cutoff value of SII in ENKTCL patients was higher than colorectal cancer, cervical cancer, and gastric cancer (SII: 601 vs. 340; 475; 508). Such difference might be due to the fact that much intense inflammatory effect was triggered in ENKTCL patients because B symptoms were common in those patients. Patients with SII-L had better OS and PFS than those with SII-H (5-year OS: 73.6% vs. 53.6%, respectively; 5-year PFS: 70.8% vs. 48.0%, respectively) (**Figure 2**). Secondly, patients with early-stage ENKTCL accounted for >70% of the population in this study, and the SII exhibited the ability to further identify patients with better survival among those included in the early-stage disease group (**Figure 3**). However, the SII did not demonstrate this ability in patients with advanced-stage disease (**Supplementary Figure 2**). The potential explanation for the lack of a prognostic effect of the SII in patients with advanced-stage disease might be the inconsistency in the treatment regimens used among these patients. Notably, the treatment of patients with advanced-stage disease was markedly more complicated than that of patients with early-stage disease. Moreover, the number of patients with advanced-stage disease was lower than that of patients with early-stage disease. This discrepancy might have impaired the integrity of the statistical analysis. As shown in **Supplementary Figure 2**, for patients with advanced-stage disease, the survival curve of the SII-L group was over that of the SII-H group. We hypothesized that the significance of the SII might gradually become apparent as the number of patients with advanced-stage disease increases. Finally, the conventional prognostic model PINK-E has the ability to stratify patients with ENKTCL into low-, intermediate-, and high-risk groups (11). In this study, the results showed that low-risk patients with SII-L have better OS and PFS than those with SII-H (**Figure 5**). The potential biological mechanisms of SII as a prognostic factor in ENKTCL might be due to the fact that strong secretion of cytokines or chemokines such as TNF-α by inflammatory cells activates immune response to suppress malignant cells. During this process, more inflammatory cells or immunocytes were mobilized, which would statistically present high SII score in patients with heavy tumor burden. Nevertheless, such significant differences in survival were not observed in the intermediate- and high-risk groups (**Supplementary Figures S4, S5**). Therefore, we suggest adding the SII as a novel predictor into the PINK-E model for the precise evaluation of prognosis in low-risk patients with ENKTCL at the time of initial diagnosis.

In addition, we established prognostic nomograms to predict the 3- and 5-year OS, as well as the 3- and 5-year PFS, of patients with ENKTCL based on Cox proportional hazards regression analysis. The parameters used to construct the nomograms included ECOG-PS score, disease stage, positivity for EBV-DNA in plasma, and the SII. The concordance index values of the nomograms for 3- and 5-year OS and PFS were 0.71 and 0.68, respectively, demonstrating a favorable predictive power. Hence, this model may assist physicians in clinical decision-making with regard to the treatment of cancer. In clinical practice, physicians specializing in the treatment of ENLTCL focus on optimizing treatment efficacy while alleviating treatment-related side effects (myelosuppression, radiation mucositis, vomiting, etc.) in patients with early-stage disease. The selection of intense or mild treatment remains a dilemma. The SII may play a critical role in facilitating this process by stratifying high- and low-risk patients to receive intense and mild treatment (e.g., lower radiation dosage and fewer chemotherapy cycles), respectively. Therefore, a multicenter prospective study focusing on the prognostic value of the SII in ENKTCL is warranted.

There were several limitations in this study. Firstly, this study was based on data obtained from a single center, without external validation data to further determine the prognostic value of the SII. Secondly, the retrospective nature of this study may have inevitably introduced selection bias and information bias. Thirdly, we did not record the dynamic fluctuation of blood parameters during treatment and follow-up, which may reflect the immune-inflammatory status.

## Conclusion

The present study is the first to demonstrate the prognostic value of the SII in patients with ENKTCL. Additionally, the SII can identify patients with better OS and PFS among those with early-stage disease or in the low-risk group in order to facilitate physicians in the selection of intense or mild treatment. Nomograms were constructed to predict the OS and PFS of patients with ENKTCL. It is recommended that the SII should be taken into consideration as a promising predictor in patients with ENKTCL.

## Data availability statement

The original contributions presented in the study are included in the article/**Supplementary Material**. Further inquiries can be directed to the corresponding author.

## Ethics statement

The studies involving humans were approved by Ethics Committee on Biomedical Research, West China Hospital of Sichuan University. The studies were conducted in accordance with the local legislation and institutional requirements. Written informed consent for participation was not required from the participants or the participants’ legal guardians/next of kin in accordance with the national legislation and institutional requirements.

## Author contributions

TH: Conceptualization, Data curation, Formal Analysis, Writing – original draft. WW: Conceptualization, Data curation, Writing – review & editing. KR: Software, Supervision, Validation, Writing – review & editing. NL: Resources, Software, Validation, Writing – review & editing. LZ: Conceptualization, Supervision, Validation, Writing – review & editing.
