# Supplementary material for: Prognostic significance of the systemic immune-inflammation index in patients with extranodal natural killer/T-cell lymphoma
Source: Front Oncol. 2023 Oct 16;13:1273504. doi: 10.3389/fonc.2023.1273504 (PMC10613892; doi:10.3389/fonc.2023.1273504)
Supplement: Supplementary file 1 [file Table_1.docx]

| **Response** | **All patients**  **(N=382) (%)** | **SII-L**  **(N=148) (%)** | **SII-H**  **(N=234) (%)** | ***p*-value** |
| --- | --- | --- | --- | --- |
| **ORR** | 286 (74.9) | 120 (81.1) | 159 (67.9) | 0.035 |
| CR | 213 (55.8) | 100 (67.6) | 113 (48.3) | 0.004 |
| PR | 73 (19.1) | 20 (13.5) | 46 (19.6) | 0.246 |
| **SD** | 16 (4.2) | 1 (0.7) | 15 (6.4) | 0.054 |
| **PD** | 80 (20.9) | 27 (18.2) | 60 (25.7) | 0.172 |

Abbreviations: CR, complete response; PR, partial response; SD, stable disease; PD, progression disease; SII-L, Systemic Immune–Inflammation Index-Low; SII-H, Systemic Immune–Inflammation Index-High.
