# Supplementary figures and images for: Prognostic significance of the systemic immune-inflammation index in patients with extranodal natural killer/T-cell lymphoma
Source: Front Oncol. 2023 Oct 16;13:1273504. doi: 10.3389/fonc.2023.1273504 (PMC10613892; doi:10.3389/fonc.2023.1273504)

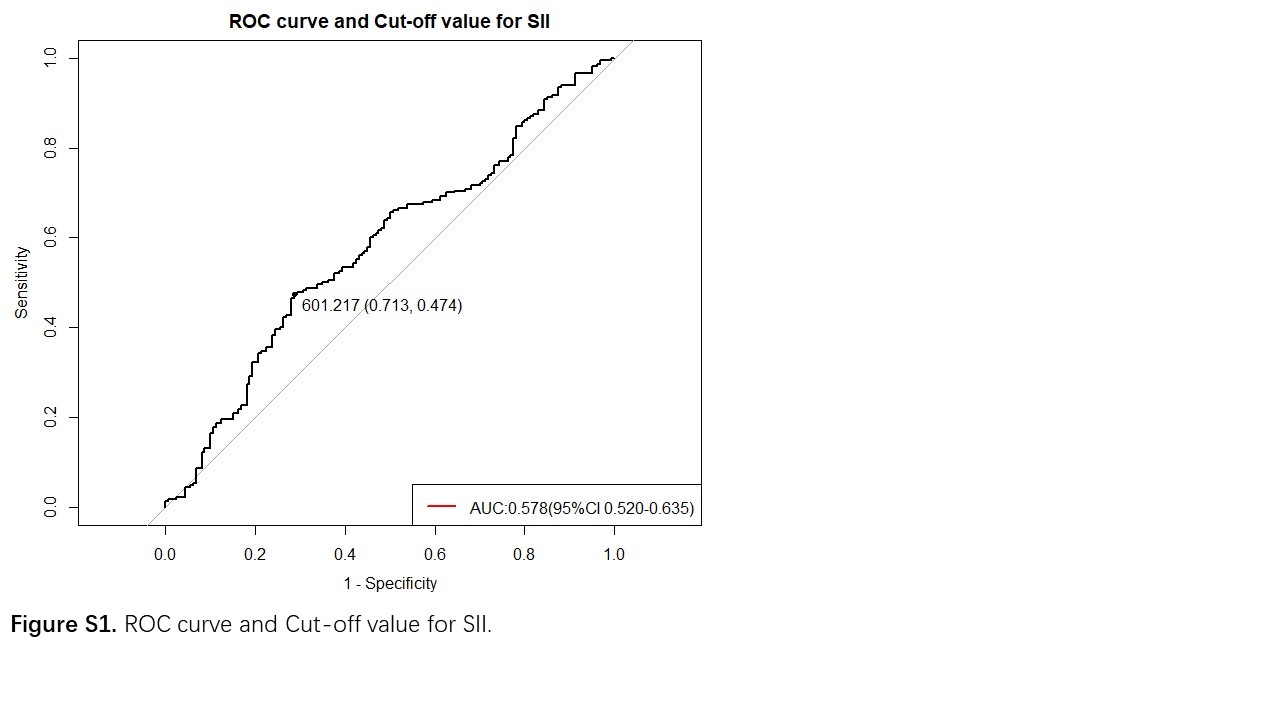

Supplement: Supplementary file 2 [file Image_1.jpeg]

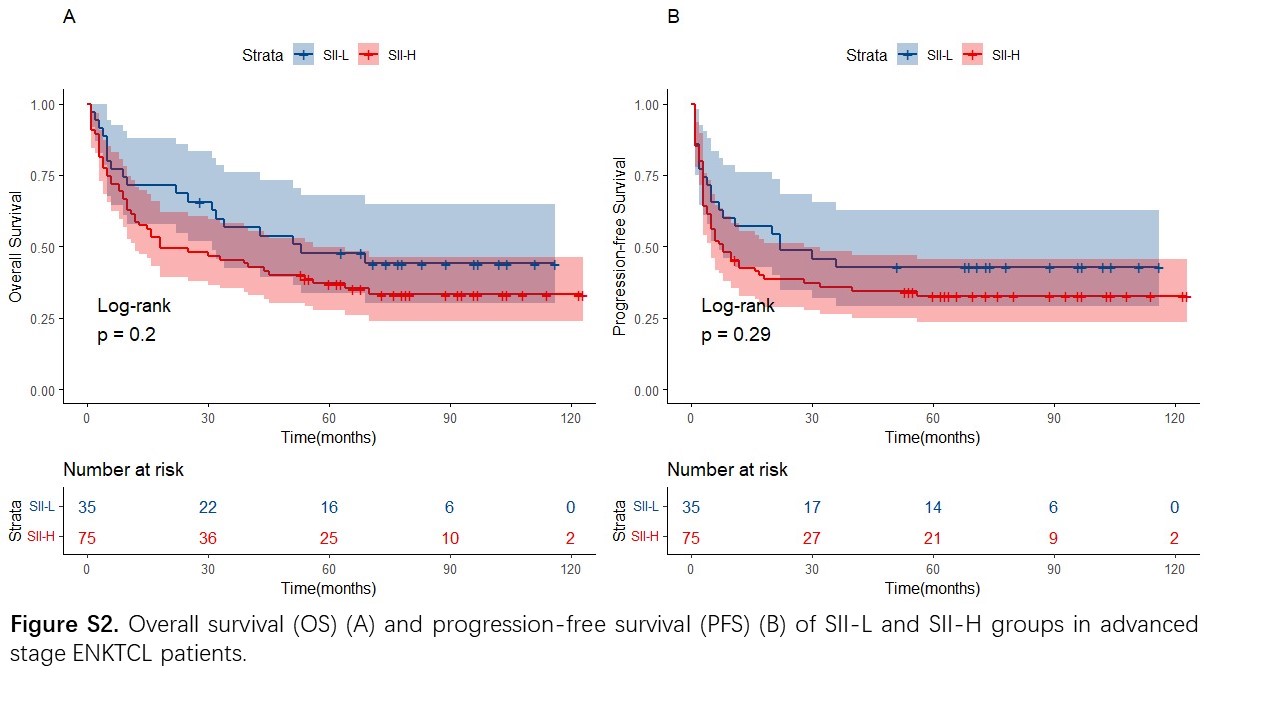

Supplement: Supplementary file 3 [file Image_2.jpeg]

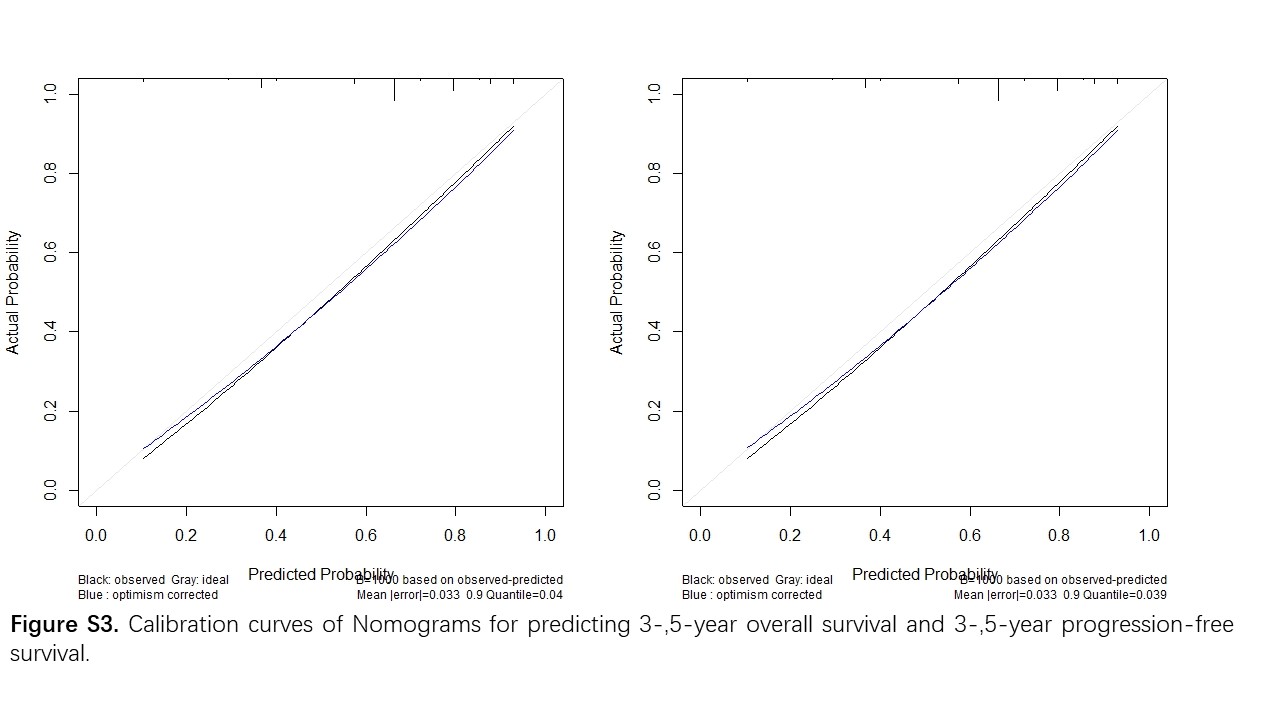

Supplement: Supplementary file 4 [file Image_3.jpeg]

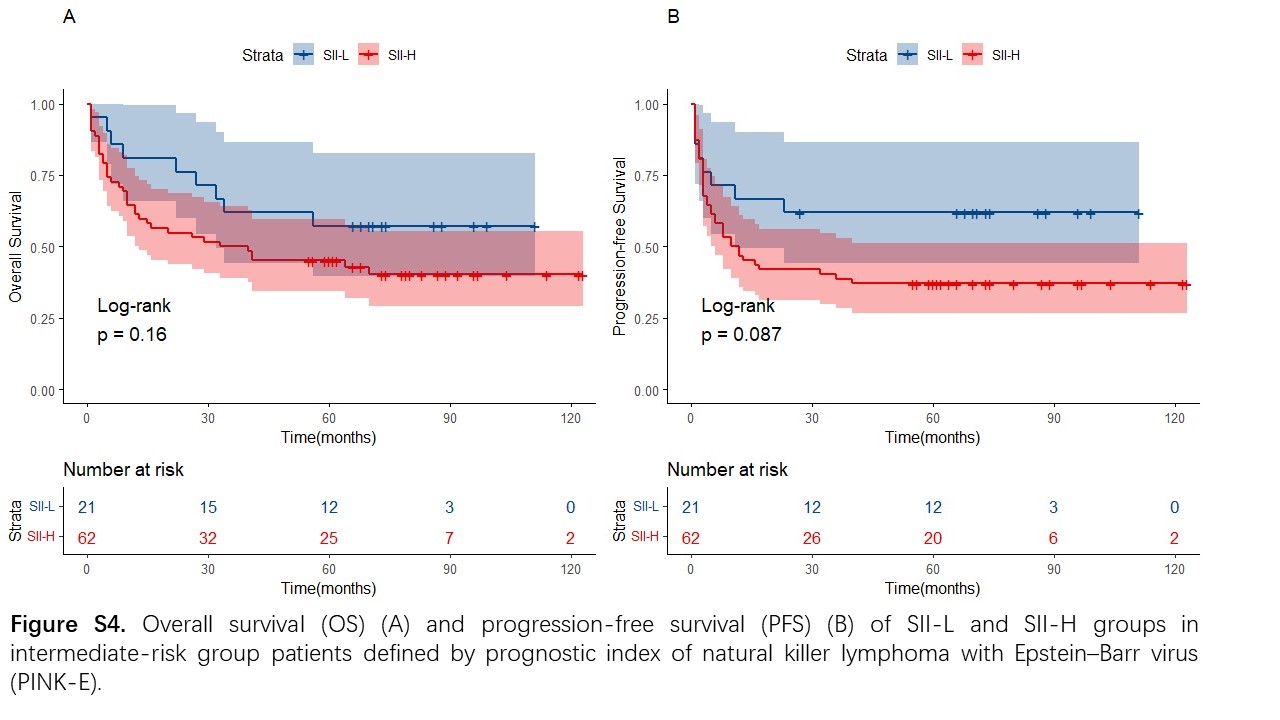

Supplement: Supplementary file 5 [file Image_4.jpeg]

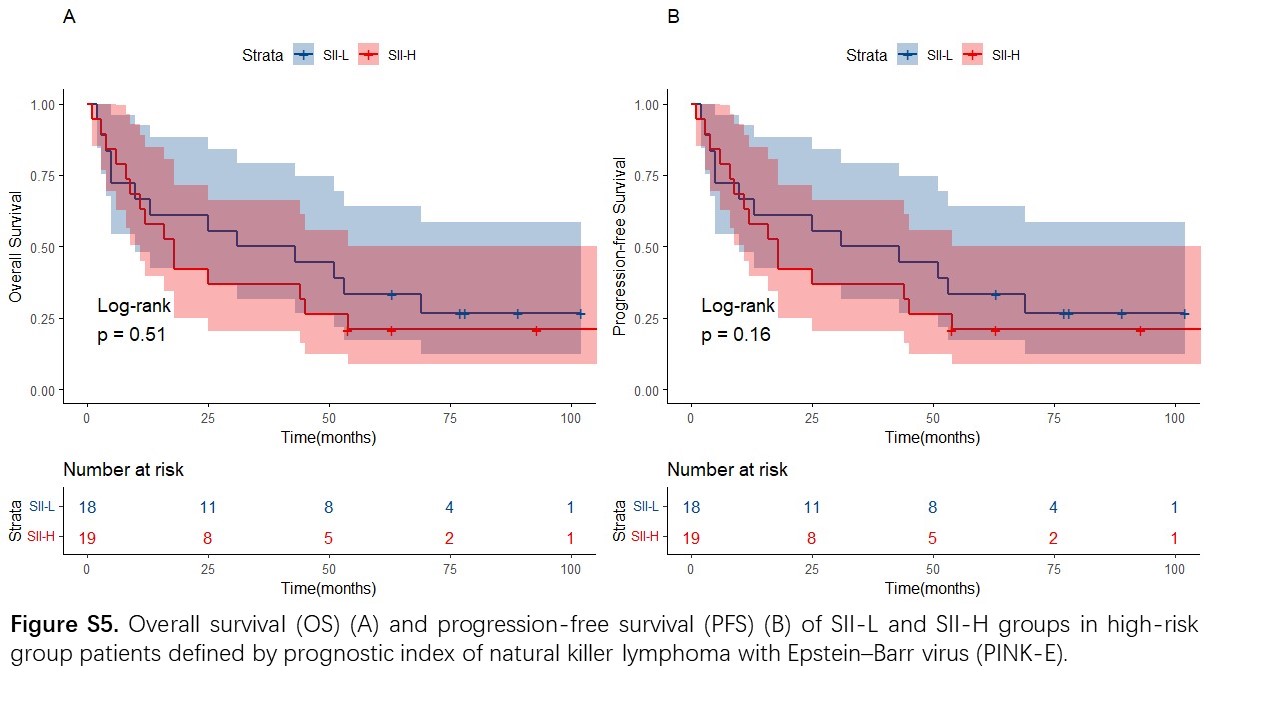

Supplement: Supplementary file 6 [file Image_5.jpeg]
